# Brain abscess and odontogenic infection

**DOI:** 10.5935/0103-507X.20200025

**Published:** 2020

**Authors:** Renata Lanzoni de Oliveira, Regina Maria Raffaele, Mario Eduardo Baldo, Ellen Cristina Gaetti Jardim

**Affiliations:** 1 Associação Beneficente Santa Casa de Campo Grande - Campo Grande (MS), Brazil.; 2 Hospital Universitário Maria Aparecida Pedrossian, Universidade Federal de Mato Grosso do Sul - Campo Grande (MS), Brazil.

**To the Editor,**

Odontogenic infections are typically limited to the dental alveolus or periodontium. However, when untreated, they can spread through fascial spaces, leading to more serious infections such as cavernous sinus thrombosis, airway obstruction, mediastinitis, endocarditis and brain abscess.^(1)^ This is explained by the proximity of the upper roots to the maxillary sinus, which can spread the infection to the ethmoid sinus, orbital cavity and brain.^(2)^ Brain abscess is a serious and potentially fatal infection. Its etiology is varied, and it may arise by bacterial dissemination from a primary lesion at a distant site or by direct contiguous invasion of a site adjacent to the infection.^(3)^ Accurate and early diagnosis is necessary, in addition to surgical intervention and high doses of antibiotics. Delayed diagnosis can lead to an unfavorable prognosis.^(4)^ Our objective is to present a case of brain abscess resulting from odontogenic infection.

**Figure 1:**
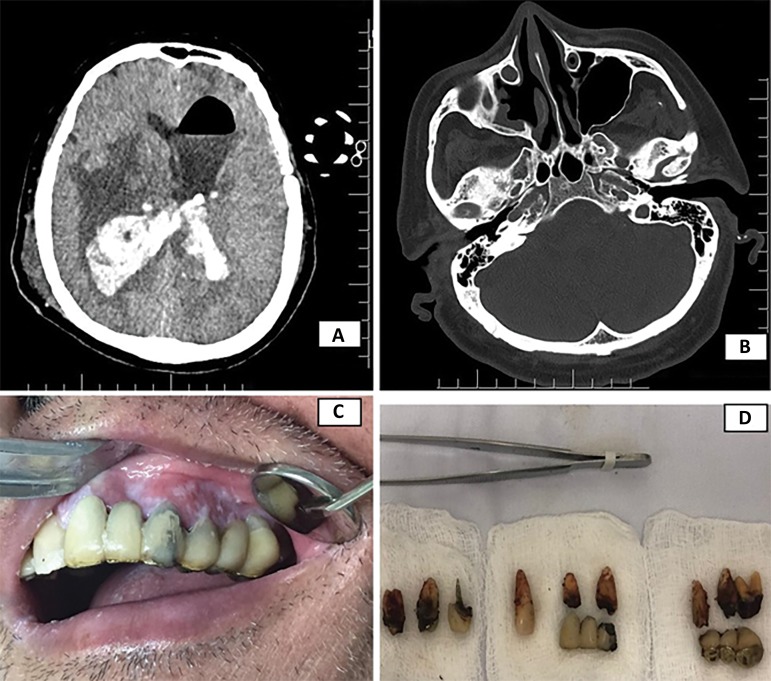
Clinical and imaging aspects of the patient with infection. (A) Computed tomography image showing a pre-existing brain injury. (B) Encapsulated regular mass and hyperdense area filling a portion of the maxillary sinus space on the right side. (C) Initial clinical appearance showing a poor state of preservation and foci of infection. (D) Dental elements after extraction.

A 60-year-old man with a history of hemorrhagic stroke, with sequelae and tetraparesis; bedridden; with chronic renal failure; not under dialysis; with a tracheostomy, a colostomy and a gastrostomy; and with a history of hospitalization due to urinary-derived sepsis was admitted to the hospital with bradycardia; hydrocephalus was also observed after computed tomography (Figures 1A and 1B). Laboratory tests showed the following: hemoglobin, 11.2g/dL; 271,000 platelets per mm^3^; 62.7 neutrophils per mm^3^; lymphocytosis (22.6 per mm^3^); international normalized ratio (INR), 0.97; and C-reactive protein (CRP), 66.3mg/dL.

Concurrently, a dental evaluation was requested, which revealed the presence of several fixed prostheses in the upper arch, with poor state of preservation and hygiene (Figure 1C and 1D). An erythematous area was present in the region of tooth 23, with swelling and fistula. Computed tomography of the skull showed an encapsulated regular mass and a hyperdense area filling a portion of the maxillary sinus space on the right side. A multidisciplinary discussion was conducted with specialists in neurosurgery, reaching the conclusion that the odontogenic infection communicating with the maxillary sinus may have led to sinusitis followed by hydrocephalus and brain abscess. Ventriculoperitoneal shunting was performed by the medical team in a surgical center under general anesthesia. Subsequently, in the intensive care unit (ICU) bed, nine dental elements in the upper arch showing infection were extracted. Propofol and morphine were used during the procedure, which resulted in a decrease in the laboratory values, especially those attributed to infection.

In cases such as this, early diagnosis is always essential for successful brain abscess treatment, and, in combination, the urgent removal of the primary source of infection cannot be avoided. The presence of the dental surgeon in the ICU setting assisted in the diagnosis and removal of the dental infection focus, in conjunction with the other specialties included in the intensive care setting.
